# Human lysozyme as a potential diagnostic marker in malaria: a mechanistic study of haemozoin-induced monocyte degranulation

**DOI:** 10.1186/1475-2875-11-S1-P80

**Published:** 2012-10-15

**Authors:** Manuela Polimeni, Elena Valente, Elisabetta Aldieri, Amina Khadjavi, Giuliana Giribaldi, Mauro Prato

**Affiliations:** 1 Dipartimento di Genetica, Biologia e Biochimica, Facoltà di Medicina e Chirurgia, Università degli Studi di Torino, Torino, 10126, Italy; 2 Dipartimento di Neuroscienze, Facoltà di Medicina e Chirurgia, Università degli Studi di Torino, Torino, 10125, Italy

## Background

Lysozymes are antibacterial proteins defined by their ability to hydrolyse beta-1,4-glycosidic linkage between N-acetylmuramic acid and N-acetylglucosamine of peptidoglycan in the cell wall of bacteria [1]. In the recent years, little evidence on their involvement in malaria pathogenesis has emerged. In *Anopheles gambiae* and *stephensi*, lysozyme was shown to bind to oocysts of *Plasmodium berghei* and *falciparum*, thereby facilitating their development within the mosquito [2]. In human patients, lysozyme plasma levels correlated significantly to parasitaemia degree, suggesting its potential role as marker of disease severity [3]. In this context, phagocytosis of haemozoin (HZ, malarial pigment) was shown in a previous work to induce *in vitro* lysozyme release from human monocytes [4]; here, the underlying mechanisms were investigated.

## Materials and methods

Human adherent monocytes from healthy donors were allowed to phagocytose for 2 h natural HZ isolated from *Plasmodium falciparum* cultures; after the end of phagocytosis, cells were incubated for 2 additional h in the presence or absence of: anti-TNFalpha/IL-1beta/MIP-1alpha blocking antibodies; recombinant TNFalpha/IL-1beta/MIP-1alpha; p38 MAPK inhibitor (SB203580); NF-kappaB inhibitors (quercetin, artemisinin, and parthenolide). Thereafter, lysozyme levels in cell supernatants were evaluated by measuring lysis of *Mycrococcus Lysodeikticus* suspensions through spectrometry, and TNFalpha, IL-1beta, and MIP-1alpha levels by ELISA. In cell lysates, p38 MAPK and NF-kappaB pathways were investigated by Western blotting or EMSA.

## Results

HZ promoted a time-dependent release of lysozyme, along with TNFalpha, IL-1beta and MlP-1alpha. HZ-induced lysozyme release was abrogated by anti-TNFalpha/IL-1beta/MIP-1alpha blocking antibodies, and mimicked by all three recombinant cytokines. Moreover, HZ early activated either p38 MAPK or NF-kappaB pathways by inducing: p38 MAPK phosphorylation; cytosolic I-kappaBalpha phosphorylation and degradation; NF-kappaB nuclear translocation and DNA-binding. Inhibition of both routes prevented HZ-dependent lysozyme release.

## Conclusions

These data suggest that the HZ-triggered overproduction of TNFalpha, IL-1beta and MlP-1alpha mediates induction of lysozyme release from human monocytes through activation of p38 MAPK and NF-kappaB pathways. Therefore, the present work provides new evidence on the mechanisms underlying HZ-enhanced monocyte degranulation in *falciparum* malaria, supporting the hypothesis that lysozyme could be used as a new affordable marker in severe malaria.
